# Recombinant proteins expressed in *Mycolicibacterium smegmatis* enhance antibody-based detection of bovine tuberculosis

**DOI:** 10.1186/s13567-026-01743-9

**Published:** 2026-05-08

**Authors:** Eomseob Jang, Kilhan Kwak, Norikazu Isoda, Kazuhiro Matsuo, Jeewan Thapa, Chie Nakajima, Yasuhiko Suzuki

**Affiliations:** 1 Hokkaido University International Institute for Zoonosis Control, Hokkaido University, Sapporo, Japan; 2 Jeonbuk State Institute of Veterinary Service and Research, Jangsu, South Korea; 3 Laboratory of Microbiology, Department of Disease Control, Faculty of Veterinary Medicine, Hokkaido University, Sapporo, Japan; 4 Institute for Vaccine Research and Development (HU-IVReD), Hokkaido University, Sapporo, Japan; 5 Bond Life Science Center, University of Missouri, Columbia, MO USA

**Keywords:** Bovine tuberculosis, *Mycobacterium bovis*, serological test, *Mycolicibacterium smegmatis*, recombinant proteins, enzyme linked immunosorbent assay (ELISA)

## Abstract

Bovine tuberculosis (bTB), caused by *Mycobacterium bovis*, is a zoonosis that threatens public health and causes substantial economic losses in livestock. The suboptimal *Escherichia coli*–expressed recombinant proteins limit the diagnostic performance of current bTB serological tests. To overcome this limitation, we evaluated *Mycolicibacterium smegmatis* as an expression host capable of producing recombinant antigens with post-translational modifications comparable to those of *M. bovis*. Ten antigen candidates were individually expressed in *E. coli* using the pET-26b( +) vector and in *M. smegmatis* using the pSOΔBam vector. Their diagnostic performance was evaluated using an enzyme-linked immunosorbent assay (ELISA) with plasma samples from interferon-gamma release assay (IGRA)-negative (*n* = 30) and -positive (*n* = 46) cattle in South Korea, followed by receiver operating characteristic (ROC) curve analysis. Among the single antigens, LprA expressed in *M. smegmatis* demonstrated diagnostic performance comparable to that of the well-established antigen MPB70 (sensitivity: 50.0%, specificity: 96.6%, AUC: 0.791). In addition, several *M. smegmatis*–derived antigens showed higher concordance with the IGRA results, as assessed by Cohen’s kappa and Fisher’s exact tests, and a stronger association between age and antigen-specific antibody responses was observed among IGRA-positive cattle. Moreover, a multiple logistic regression model incorporating eight antigens, including those derived from both hosts, achieved high predictive accuracy for IGRA results (sensitivity: 87.0%, specificity: 100%, AUC: 0.991). These findings suggest that *M. smegmatis* is a promising host for identifying novel antigens and that a multi-host strategy may improve bTB serodiagnosis.

**Supplementary Information:**

The online version contains supplementary material available at 10.1186/s13567-026-01743-9.

## Introduction

Tuberculosis (TB), caused by the *Mycobacterium tuberculosis* complex (MTBC), is a major infectious disease and a persistent threat to global public health [1]. Within the MTBC, *Mycobacterium bovis* is the primary causative agent of bovine tuberculosis (bTB), which affects animal health and imposes substantial economic burdens on livestock industries. According to the World Organization for Animal Health (WOAH), bTB was reported in 82 of 188 countries (44%) between 2017 and 2018 [2]. Globally, bTB is estimated to result in annual economic losses of approximately USD 3 billion [3]. Beyond its economic impact on agriculture, bTB is also an important zoonotic disease. Human infection with *M. bovis,* known as zoonotic tuberculosis (zTB), primarily occurs through the consumption of unpasteurized dairy products. zTB remains a serious public health concern, especially in low- and middle-income countries, where practical bTB control efforts are often hindered by economic and cultural constraints [4]. In 2019, zTB accounted for approximately 140 000 new infections and 11 400 deaths worldwide [5]. Importantly, sporadic cases of zTB continue to occur even in high-income countries with advanced public health systems. Between 2019 and 2023, the European Union reported an annual average of over 122 human *M. bovis* infections [6]. Moreover, South Korea confirmed its first zTB case, involving a laboratory worker exposed to animal specimens, highlighting the ongoing occupational risks associated with *M. bovis* transmission [7].

To mitigate the economic and public health burden of bTB, many countries have implemented antemortem diagnostic tests in cattle, including the tuberculin skin test (TST) and interferon-gamma release assay (IGRA), both of which assess cell-mediated immune responses [8]. For example, South Korea conducts approximately 230 000 TSTs and 750 000 IGRAs annually, costing around USD 4.2 million. Likewise, the United Kingdom allocates approximately GBP 120 million annually to bTB control efforts [9]. Despite these efforts, bTB remains endemic in many regions, likely because of the continued movement of infected cattle, limited diagnostic sensitivity (DSe) and specificity (DSp), and the existence of environmental and wildlife reservoirs [10].

Although TST and IGRA remain central to current bTB control strategies, financial and logistical challenges have spurred interest in alternative diagnostics. The progressive increase in antibody titers during bTB infection, alongside advances in *M. bovis* genome sequencing [11] and recombinant protein expression in *E. coli* [12], has facilitated the development of serological diagnostics targeting *M. bovis*–specific antigens [13–17]. These tests can identify animals that yield false-negative results on TST or IGRA and offer high DSp by minimizing cross-reactivity with environmental non-tuberculous mycobacteria (NTM) [14–16]. However, their broader application is limited by delayed seroconversion and lower DSe compared with cell-mediated immunity (CMI)-based assays [18, 19]. Recent large-scale screenings of over 100 recombinant antigens have revealed marked heterogeneity in antibody responses among *M. bovis*–infected cattle, highlighting the challenges of identifying optimal diagnostic antigens [20]. This heterogeneity may stem from the inability of *E. coli* expression systems to recapitulate the post-translational modifications (PTMs) present in native *M. bovis* proteins.

PTMs involve the covalent addition of functional groups such as phosphates, lipids, or carbohydrates. These modifications are present in *M. tuberculosis* proteins, and they modulate protein structure and host immune responses [21, 22]. Enhanced antibody reactivity to native *M. tuberculosis* proteins, compared with their *E. coli*–expressed recombinant counterparts, highlights the diagnostic relevance of PTMs in patients with TB [23]. However, the high pathogenicity and slow growth of major MTBC species, including *M. bovis* and *M. tuberculosis*, limit their use for large-scale antigen production. To overcome these limitations, *Mycolicibacterium smegmatis*, a non-pathogenic and fast-growing mycobacterial species that is phylogenetically close to pathogenic mycobacteria, has emerged as a promising alternative host [24, 25]. This expression host supports the production of soluble, properly folded, and biologically active mycobacterial proteins [26]. In addition, it is well established that proteins expressed in *M. smegmatis* undergo PTMs comparable to those of native pathogens, which facilitates a more accurate evaluation of their immunogenicity [27–30]. Based on these advantages, we hypothesized that recombinant proteins expressed in *M. smegmatis* would improve the diagnostic performance of serological tests for bTB.

In this study, we evaluated the diagnostic performance of ten antigen candidates expressed in *E. coli* and *M. smegmatis* by enzyme-linked immunosorbent assay (ELISA) using plasma samples from IGRA-negative and -positive cattle. By comparing the antigenicity of proteins from each expression system, we aimed to clarify the influence of PTMs on serological diagnostic performance, thereby contributing to improved bTB diagnostic strategies.

## Materials and methods

### Plasma sample collection 

Between 2022 and 2024, whole blood samples were collected from eight cattle farms in Jeonbuk, South Korea, as part of standard veterinary clinical practice. Plasma was isolated at the Jeonbuk State Institute of Veterinary Service and Research for IGRA testing in accordance with guidelines issued by the Ministry of Agriculture, Food and Rural Affairs [31]. To avoid potential confounding effects of antigen stimulation on ELISA performance, only plasma obtained from PBS-treated (unstimulated) whole blood was used in this study.

### Interferon-gamma release assay and MAP antibody ELISA

IGRA was performed using the PIron-gamma TB ELISA Kit (PIgenomics Co., Ltd., Hwaseong, Korea) according to the manufacturer’s instructions, with synthetic peptides of early secreted antigenic target 6 (ESAT-6), culture filtrate protein 10 (CFP-10), and the low-molecular-weight antigen TB10.4 (EsxH) serving as stimulating antigens. For the IGRA-negative samples, a *Mycobacterium avium* subsp. *paratuberculosis* (MAP) antibody ELISA was conducted using the Johne’s Disease Screening KS kit (Kyoritsu Seiyaku Corp., Tokyo, Japan) to evaluate potential MAP-associated humoral responses that could confound antibody reactivity to the recombinant antigens. In this assay, plates were coated with purified proteins from the *M. avium* subsp. *avium* P-18 strain [32]. Following the manufacturer's protocol, plasma samples were pre-adsorbed with *Mycobacterium phlei* strain 354-NIAH prior to the ELISA to ensure specificity. No MAP-seropositive samples were detected among IGRA-negative animals.

### Classification of IGRA samples

The IGRA-negative group (*n* = 30) comprised samples from five farms without reported bTB cases during the 5-year period (2020–2024). A conservative selection approach was applied, in which only samples exhibiting OD differences (antigen-stimulated minus PBS control) ≤ 0.01 were included.

The IGRA-positive group (*n* = 46) from three farms consisted of samples exhibiting OD differences ≥ 0.1, as defined by the manufacturer’s guidelines. To represent a broad range of IGRA reactivity, the group was subdivided into high (OD ≥ 1.0, *n* = 8), moderate (0.3 ≤ OD < 1.0, *n* = 16), and low (0.1 ≤ OD < 0.3, *n* = 22) responder categories. Adequate mitogen responses were confirmed for all samples, ensuring sufficient T-cell activation. A complete list of sample information is provided in Additional file 1.

### Production of recombinant antigen candidates in *E. coli* and *M. smegmatis*

Ten antigen candidates, including the chimeric ESAT-6/CFP-10 protein (E6C10), were selected based on their reported immunogenicity in humans and cattle, their abundance in bovine purified protein derivative (bPPD), known PTMs, and potential cross-reactivity with MAP [13, 33]. The characteristics of these antigen candidates are summarized in Table 1.

**Table 1:** **Characteristics of antigen candidates included in this study**

Mb locus	Protein name	Reported PTMs in M. bovis*	Abundance (%) in bPPD [33]	Similarity (%) with MAP proteins**
Mb1961c	Immunogenic protein MPB63	–	1.5	46
Mb2002c	Immunogenic protein MPB64	–	0.9	55
Mb2900	Immunogenic protein MPB70	–	5.0	nd
Mb2898	Cell surface glycolipoprotein MPB83	G, L	0.5	nd
Mb1301c	Putative lipoprotein LprA	G, L	0.4	41
Mb1403	Putative diacylated glycolipid transporter LprF	G, L	0.1	30
Mb1446c	Lipoarabinomannan carrier protein LprG	G, L	0.2	68
Mb2970c	Putative phthiocerol dimycocerosate transporter LppX	G, L	0.4	35
Mb3789	Lipoprotein LpqH	G, L	0.1	76
Mb3905	Early secreted antigenic target of 6 kDa	A	15	34
Mb3904	10 kDa culture filtrate antigen CFP-10	–	35	34

PTMs, post-translational modifications; bPPD, bovine purified protein derivative; MAP, *Mycobacterium avium* subsp.* paratuberculosis.*

^*^G: glycosylation, L: lipidation, A: acetylation.

^**^Protein BLAST was performed using the standard database and BLASTp algorithm on NCBI. If the query sequence was not identified in *Mycobacterium avium subsp. paratuberculosis (taxid:1770)*, it was denoted as “nd” (not detected).

For *E. coli* expression, primers containing restriction enzyme sites and a 6 × histidine tag were designed to amplify each target gene from *M. bovis* BCG Tokyo 172 genomic DNA (Japan BCG Laboratory, Tokyo, Japan) using PCR with TaKaRa LA Taq polymerase (Takara Bio Inc., Shiga, Japan). The resulting amplicons and the pET‑26b(+) vector (Novagen, Merck KGaA, Darmstadt, Germany) were digested with the corresponding restriction enzymes (New England Biolabs, Ipswich, MA, USA), ligated, and transformed into *E. coli* BL21(DE3)pLysS cells via heat shock (42 °C, 45 s). Transformed *E. coli* cells were cultured in Terrific Broth (BD Difco, Franklin Lakes, NJ, USA) to an OD_590_ of 0.6–1.0, after which protein expression was induced with isopropyl β-D-thiogalactopyranoside at optimized concentrations and temperatures. Following induction, cells were harvested by centrifugation (5000 × *g*, 30 min), resuspended in Native Binding Buffer (50 mM NaH₂PO₄, 500 mM NaCl, pH 8.0), and lysed by sonication (30 s on/off for 10 min, medium power). The lysate was centrifuged (12 000 × *g*, 10 min), and soluble fractions were collected and purified using Ni–NTA agarose (Thermo Fisher Scientific, Waltham, MA, USA) with a gradient of 0 to 250 mM imidazole. The codon-optimized E6C10 gene (GenScript Biotech, Piscataway, NJ, USA) was synthesized, subcloned into pET-26b(+), and processed using the same expression and purification workflow.

For *M. smegmatis* expression, codon-optimized genes (MPB63, MPB64, MPB70, MPB83, and E6C10; GenScript Biotech) or PCR products from *M. bovis* BCG Tokyo 172 genomic DNA (for the remaining antigens) were cloned into the expression vector pSOΔBam and electroporated into *M. smegmatis* strains ATCC 607 or mc^2^ 155 using a NEPA Porator with a single pulse at 2000 V (Nepa Gene Co., Ltd., Ichikawa, Japan). Transformants were plated on LB agar and incubated at 37 °C for 3 days, after which single colonies were cultured in Sauton’s medium at 37 °C for at least 7 days [34]. Culture supernatants were filtered through 0.2 µm Minisart syringe filters (Sartorius AG, Göttingen, Germany) and purified using Ni‑NTA agarose (Thermo Fisher Scientific) with a gradient of 0 to 250 mM imidazole. Biofilms were resuspended in PBS containing 0.05% Tween 80, centrifuged (10 000 × *g*, 30 min), and processed using the same purification workflow as for *E. coli* lysates.

Protein molecular weight and purity were assessed by SDS‑PAGE, and protein expression was confirmed by western blot using anti-His-tag mAb-HRP-DirecT (MBL Life Science, Tokyo, Japan). Representative SDS-PAGE results are shown in Additional file 2. All recombinant proteins were concentrated using Amicon ultra centrifugal filter units (Merck KGaA, Darmstadt, Germany), subjected to buffer exchange into PBS using PD‑10 desalting columns (Cytiva, Marlborough, MA, USA), and stored at −80 °C until use. Primer sequences, restriction enzymes, and detailed culture conditions are provided in Additional files 3 and 4.

### In-house ELISA

Indirect ELISA was performed in triplicate on 96‑well Maxisorp plates (Thermo Fisher Scientific) coated with 1 µg/mL antigen in carbonate buffer (15 mM Na₂CO₃, 35 mM NaHCO₃, 0.05% NaN₃, pH 9.6) for 1 h at 25 °C. After three washes with PBS-T (0.05% Tween 20 in PBS), plates were blocked with blocking buffer (Block ACE, Megmilk Snow Brand Co., Ltd., Sapporo, Japan) for 1 h at 25 °C, followed by three additional washes with PBS-T. Plasma samples diluted 1:500 in blocking buffer were added and incubated for 1 h at 25 °C. After PBS-T washes, HRP-conjugated rabbit anti-bovine IgG (1:20 000 dilution in PBS; Sigma-Aldrich, St. Louis, MO, USA) was added and incubated for 1 h at 25 °C. The colorimetric reaction was developed using TMB substrate (KPL SureBlue TMB Microwell Peroxidase Substrate, 1-Component; SeraCare, Milford, MA, USA) for 15 min in the dark at 25 °C. Reactions were stopped with 1 M phosphoric acid, and absorbance was measured at 450 nm using a microplate reader (SpectraMax 190, Molecular Devices, San Jose, CA, USA). Background absorbance from no‑plasma wells was subtracted before analysis.

### Statistical analysis

ELISA data were analyzed using Prism 9 (GraphPad Software, San Diego, CA, USA) and Python version 3.13 with the pandas, numpy, and scikit-learn libraries. Differences in OD values between IGRA-negative and -positive groups were assessed using the Mann–Whitney U test, with a two-tailed *p*-value < 0.05 considered statistically significant.

The diagnostic performance of each recombinant antigen was evaluated using receiver operating characteristic (ROC) curve analysis based on IGRA status, and 95% confidence intervals (CIs) were determined using the Wilson/Brown method. For all 20 antigens, the area under the curve (AUC), DSe, and DSp were calculated. The statistical significance of the differences in the AUCs for each antigen between expression hosts was evaluated using DeLong's test, which was performed using R software (version 4.5.1; R Foundation for Statistical Computing, Vienna, Austria) with the pROC package.

Cohen’s kappa and Fisher’s exact tests were used to examine the agreement and statistical association, respectively, between binary ELISA classifications and IGRA status for each antigen. ELISA cut-off values were defined as the mean OD of IGRA-negative samples plus three standard deviations (SD). The relationship between cattle age and antibody reactivity for each antigen was evaluated using Spearman’s rank correlation coefficient.

A multiple logistic regression (MLR) model was constructed using ELISA data from all 20 antigens across 76 plasma samples. OD values for each antigen served as independent variables, and IGRA status (positive/negative) as the dependent variable. Forward stepwise selection was employed to identify combinations of antigens expressed in *E. coli*, *M. smegmatis*, or both, while minimizing multicollinearity, defined as a variance inflation factor (VIF) < 10. Model performance was assessed based on goodness-of-fit, statistical significance of predictors, and classification accuracy, including ROC curve analysis, using the fitted MLR model, with full results provided in Additional files 5–7. In addition, internal validation was performed using bootstrap resampling (B = 1000). Model discrimination was calculated using Somers’ Dxy and converted to the AUC as AUC = (Dxy + 1)/2 with the rms package in R (version 4.5.1).

## Results

### Evaluation of individual discriminative and diagnostic performance of recombinant proteins expressed in *E. coli* and *M. smegmatis*

**Figure 1:**
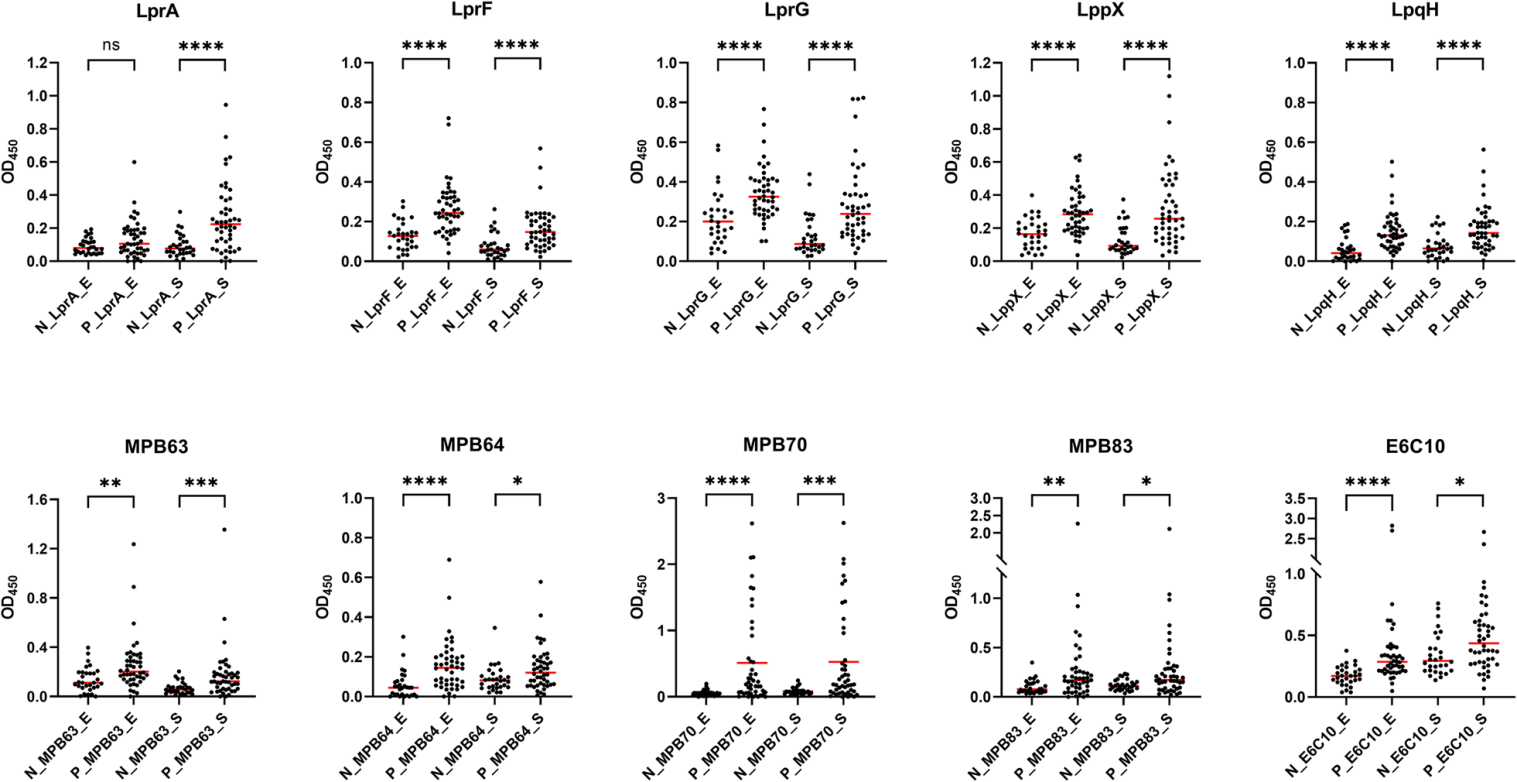
**Comparison of antigen‑specific IgG responses between interferon-gamma release assay (IGRA)‑positive and -negative groups.** Dot plots show ELISA absorbance (OD_450_) for each recombinant protein: ***N_x_E*** and ***P_x_E*** indicate IGRA‑negative (n = 30) and IGRA‑positive (n = 46) plasma samples tested against *E. coli*-expressed proteins, respectively. ***N_x_S*** and ***P_x_S*** denote the same groups tested against *M. smegmatis*‑expressed proteins. Red horizontal lines represent median OD values. Statistical significance between the groups is shown as: ns (not significant), *p < 0.05, **p < 0.01, ***p < 0.001, ****p < 0.0001.

Ten antigen candidates were individually expressed in *E. coli* and *M. smegmatis*, resulting in 20 recombinant proteins, designated with the suffixes _E or _S to indicate the respective expression hosts. For each antigen, ELISA OD values were compared between IGRA‑negative (*n* = 30) and IGRA‑positive (*n* = 46) groups (Figure 1). Nineteen of the 20 recombinant proteins showed statistically significant group differences (*p* < 0.05). Four proteins, namely LprF, LprG, LppX, and LpqH, showed strong group separation between IGRA-negative and IGRA-positive cattle regardless of expression host (*p* < 0.0001). Likewise, LprA_S, MPB64_E, MPB70_E, and E6C10_E exhibited highly significant group differences (*p* < 0.0001). In contrast, only LprA_E failed to exhibit a statistically significant difference (*p* > 0.11).

**Figure 2:**
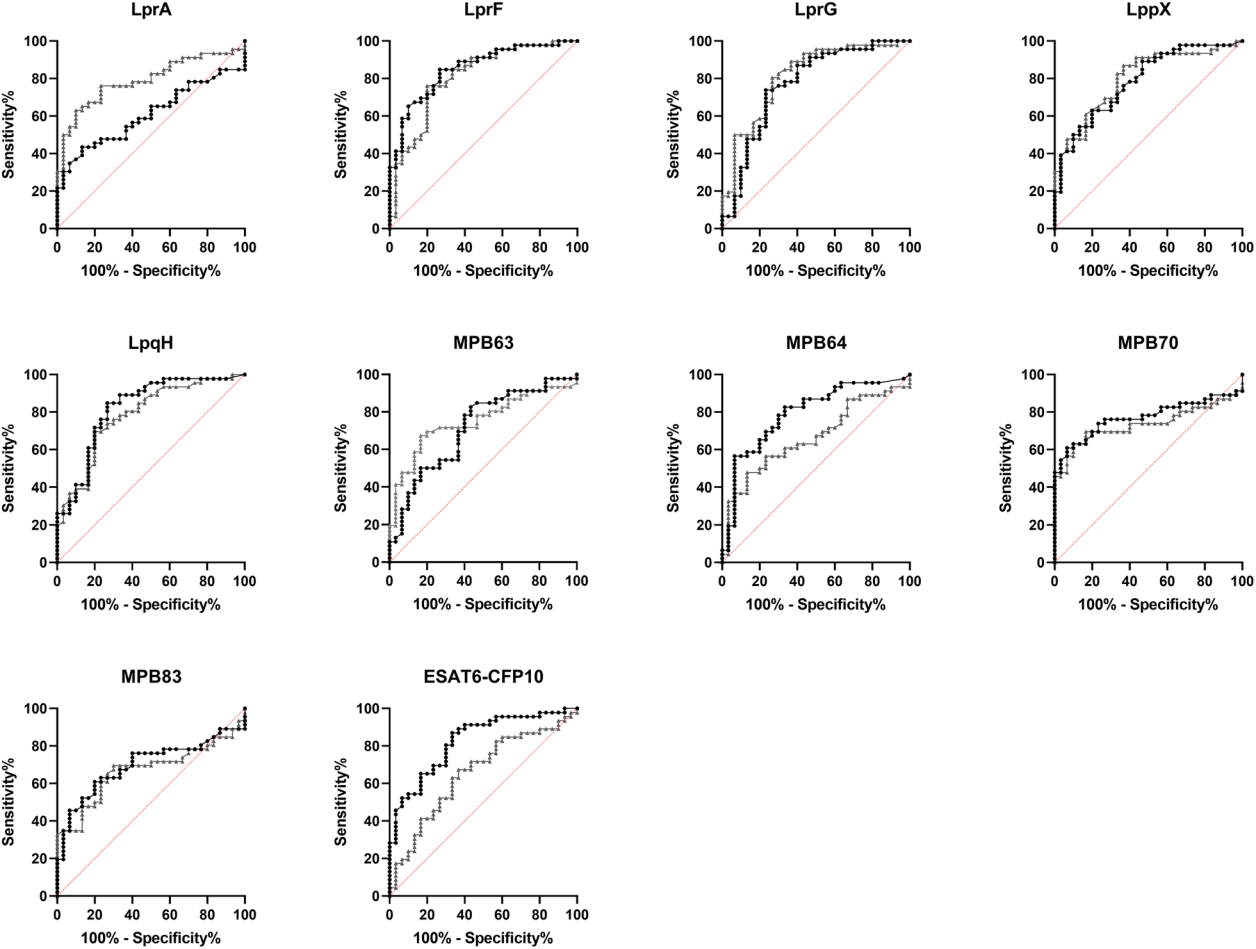
**Diagnostic performance of 20 antigen candidates evaluated by ROC curve analysis.** Black and gray curves indicate recombinant proteins produced in *E. coli* and *M. smegmatis*, respectively. In each plot, the red dashed line represents the reference line (AUC = 0.5), indicating no discriminatory ability.

ROC curve analysis was performed to determine the AUC for each antigen based on IGRA status (Figure 2 and Table 2). To minimize non-specific ELISA reactivity among IGRA-negative cattle, DSe was evaluated at a cut-off corresponding to a DSp of 96.6%. Among the 20 recombinant proteins, MPB70_E yielded the highest DSe (54.4%), whereas LprG_E showed the lowest (6.52%). For proteins expressed in *M. smegmatis*, LprA_S and E6C10_S exhibited the highest (50.0%) and lowest (17.4%) DSe, respectively. Six antigens (LprF_E, LpqH_E, E6C10_E, LprF_S, LprG_S, and LppX_S) exhibited AUC values ≥ 0.8. The highest AUC (0.852) was observed for LprF_E, and the lowest (0.608) for LprA_E.

**Table 2:** **Diagnostic performance of individual antigen candidates based on ROC curve analysis**

Name	Expression Host	OD^450^^†^	DSe (%)	95% CI	AUC	Delong’s test^‡^ (*p*-value)
LprA	*E. coli*	> 0.182	30.4	19.1–44.8	0.608	< 0.01 (*)
	*M. smegmatis*	> 0.225	50.0	36.1–63.9	0.791	
LprF	*E. coli*	> 0.278	41.3	28.3–55.7	0.852	0.29 (ns)
	*M. smegmatis*	> 0.200	34.8	22.7–49.2	0.809	
LprG	*E. coli*	> 0.573	6.52	2.24–17.5	0.770	0.36 (ns)
	*M. smegmatis*	> 0.408	19.6	10.7–33.2	0.815	
LppX	*E. coli*	> 0.303	39.1	26.4–53.5	0.786	0.72 (ns)
	*M. smegmatis*	> 0.310	39.1	26.4–53.5	0.802	
LpqH	*E. coli*	> 0.185	26.1	15.6–40.3	0.822	0.26 (ns)
	*M. smegmatis*	> 0.194	30.4	19.1–44.8	0.785	
MPB63	*E. coli*	> 0.354	13.0	6.12–25.7	0.715	0.38 (ns)
	*M. smegmatis*	> 0.164	41.3	28.3–55.7	0.753	
MPB64	*E. coli*	> 0.217	19.6	10.7–33.2	0.794	< 0.01 (*)
	*M. smegmatis*	> 0.169	32.6	20.9–47.0	0.666	
MPB70	*E. coli*	> 0.158	54.4	40.2–67.9	0.771	0.02 (*)
	*M. smegmatis*	> 0.206	47.8	34.1–61.9	0.736	
MPB83	*E. coli*	> 0.214	34.8	22.7–49.2	0.699	0.44 (ns)
	*M. smegmatis*	> 0.231	34.8	22.7–49.2	0.671	
E6C10	*E. coli*	> 0.303	45.7	32.2–59.8	0.827	0.01 (*)
	*M. smegmatis*	> 0.764	17.4	9.09–30.7	0.657	

DSe, Diagnostic sensitivity; DSp, Diagnostic specificity; CI, confidence interval; AUC, area under the curve

^**†**^An OD_450_ cut-off value was determined to achieve a DSp of at least 96.6%

^‡^Significance levels: *p* < 0.05 (*); ns, not significant

Differences in the AUCs between expression hosts for each antigen pair were assessed using DeLong’s test (Table 2). Statistically significant differences were observed for LprA, MPB64, MPB70, and E6C10 (*p* < 0.05). LprA_S, MPB64_E, MPB70_E, and E6C10_E demonstrated higher discriminative ability compared to their respective counterparts in ROC-based analysis, whereas no significant differences were observed for the remaining antigen pairs.

Agreement between ELISA results for each antigen and IGRA status was evaluated using Cohen’s kappa (κ) coefficient, with statistical association further examined by Fisher’s exact test (Table 3). Fourteen of the 20 antigens showed statistically significant Cohen’s kappa statistics when compared with the IGRA results, indicating slight-to-fair agreement. Regardless of expression host, the highest agreement was observed for MPB70_E (κ = 0.393) and MPB70_S (κ = 0.372). Five antigens (LprA_S, LppX_S, MPB70_E, MPB70_S, and MPB83_S) showed fair agreement (κ = 0.21–0.40), whereas nine antigens, including LprA_E, LprF_E, LprG_S, LppX_E, LpqH_E, LpqH_S, MPB63_S, MPB83_E, and E6C10_E, showed slight agreement (κ = 0.00–0.20). In the comparison of expression hosts, higher κ values were generally observed for LprF and E6C10 when expressed in *E. coli*, whereas MPB63, MPB83, LprA, and LppX showed higher agreement when expressed in *M. smegmatis*. Consistent with the κ-based analysis, Fisher’s exact test identified statistically significant associations for 10 antigens, including five expressed in *E. coli* (LprF_E, LppX_E, LpqH_E, MPB70_E, and E6C10_E) and five expressed in *M. smegmatis* (LprA_S, LppX_S, MPB63_S, MPB70_S, and MPB83_S).

**Table 3:** **Agreement between antigens and IGRA results, and correlations between cattle age (month) and ELISA results**

Name	Expression Host	Cohen’s κ^†^ (95% CI)	Fisher’s exact test *p*-value^†^	Spearman r^‡^ (95% CI, *p*-value)
LprA	*E. coli*	0.106 (0.020–0.191)	ns	0.402 (0.114–0.627, **)
	*M. smegmatis*	0.249 (0.107–0.392)	**	0.493 (0.225–0.692, ***)
LprF	*E. coli*	0.161 (0.056–0.266)	**	0.436 (0.154–0.652, **)
	*M. smegmatis*	0.026 (− 0.052 to 0.103)	ns	0.541 (0.286–0.724, ***)
LprG	*E. coli*	0.035 (−0.014–0.083)	ns	0.307 (0.006–0.557, *)
	*M. smegmatis*	0.116 (0.006–0.226)	ns	0.558 (0.308–0.736, ****)
LppX	*E. coli*	0.124 (0.032–0.217)	*	0.549 (0.296–0.730, ****)
	*M. smegmatis*	0.257 (0.126–0.388)	***	0.601 (0.366–0.764, ****)
LpqH	*E. coli*	0.124 (0.032–0.217)	*	0.582 (0.340–0.752, ****)
	*M. smegmatis*	0.106 (0.020–0.191)	ns	0.565 (0.318–0.741, ****)
MPB63	*E. coli*	0.052 (− 0.007 to 0.112)	ns	0.274 (− 0.030 to 0.532, ns)
	*M. smegmatis*	0.161 (0.056–0.266)	**	0.405 (0.118–0.630, **)
MPB64	*E. coli*	0.079 (− 0.019 to 0.177)	ns	0.313 (0.013–0.562, *)
	*M. smegmatis*	0.061 (− 0.030 to 0.153)	ns	0.318 (0.018–0.565, *)
MPB70	*E. coli*	0.393 (0.230–0.555)	****	0.453 (0.175–0.664, **)
	*M. smegmatis*	0.372 (0.211–0.532)	****	0.462 (0.187–0.671, **)
MPB83	*E. coli*	0.134 (0.019–0.250)	ns	0.368 (0.075–0.603, *)
	*M. smegmatis*	0.237 (0.111–0.364)	**	0.380 (0.088–0.611, *)
E6C10	*E. coli*	0.199 (0.082–0.315)	**	0.540 (0.284–0.724, ***)
	*M. smegmatis*	0.008 (− 0.062 to 0.078)	ns	− 0.055 (− 0.351 to 0.251, ns)

CI, confidence interval

^**†**^ELISA results were classified as positive if the OD value exceeded the mean OD of the IGRA-negative group plus three standard deviations (mean + 3SD) for Cohen’s κ and Fisher’s exact test

^‡^Spearman’s rank correlation was calculated between each antigen’s ELISA OD value and the ages (in months) of 45 IGRA-positive cattle with confirmed individual identification numbers

Significance levels: *p* < 0.05 (*); *p* < 0.01 (**); *p* < 0.001 (***); *p* < 0.0001 (****); ns, not significant

The association between age and antibody reactivity was assessed using Spearman’s rank correlation analysis based on ELISA OD values for each antigen and the age (in months) of 45 IGRA-positive cattle with individual identification numbers (Table 3 and Additional file 1). Among the 20 antigens analyzed, statistically significant correlations between age and antibody reactivity were observed for 18 antigens (*p* < 0.05), excluding MPB63_E and E6C10_S. Seven antigens (LprF_S, LprG_S, LppX_E, LppX_S, LpqH_E, LpqH_S, and E6C10_E) showed a moderate positive correlation (ρ ≥ 0.5), whereas the remaining 11 antigens exhibited a weak positive correlation (0.3 ≤ ρ < 0.5).

### Optimized recombinant protein combinations derived from *E. coli* and *M. smegmatis* enhance diagnostic performance

**Figure 3:**
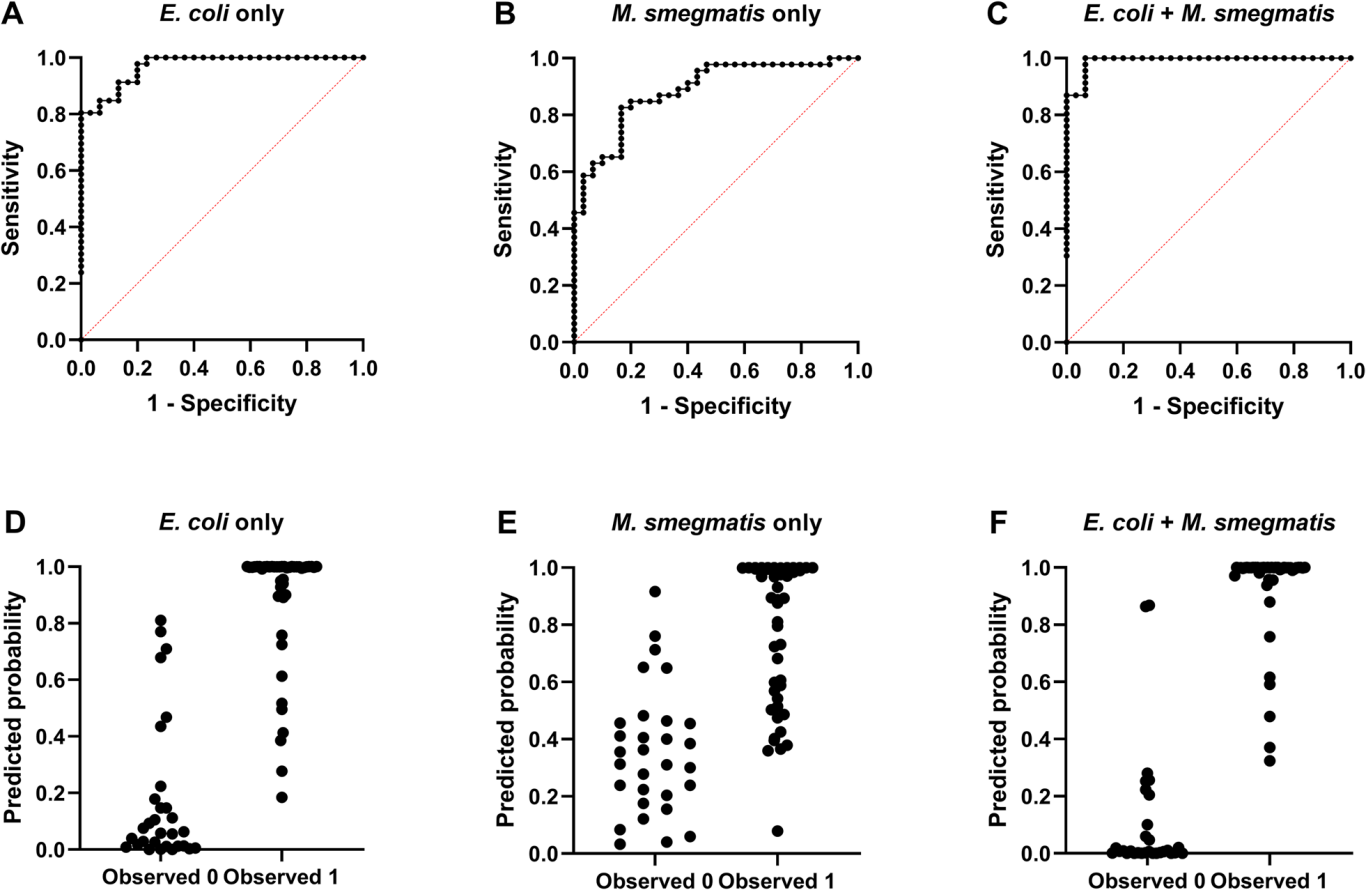
**Performance of three multiple logistic regression models based on recombinant antigens for IGRA classification.** (**A**–**C)** Receiver operating characteristic (ROC) curves of three logistic regression models showing concordance with IGRA classifications. Black dotted lines represent the ROC curves of individual models , and the red dashed line indicates random classification (AUC = 0.5). (**D–F)** Predicted vs. observed plots illustrating the distribution of predicted probabilities for IGRA-positive (Observed = 1) and -negative (Observed = 0) samples.

Three MLR models were constructed using *E. coli*–derived proteins, *M. smegmatis*–derived proteins, and their combination to assess the diagnostic performance of multi-antigen panels (Figure 3, Table 4, and Additional files 5–7). Among the *E. coli*–derived combinations without multicollinearity (VIF < 10), the panel comprising eight antigens (LprA_E, LprF_E, LprG_E, LpqH_E, MPB63_E, MPB64_E, MPB70_E, and E6C10_E) achieved the highest AUC (0.970), with a DSe of 80.4% and a DSp of 100.0% at a cut-off of 0.820. In contrast, the optimal *M. smegmatis*–derived combination, comprising six antigens (LprF_S, LprG_S, MPB63_S, MPB64_S, MPB70_S, and MPB83_S), yielded an AUC of 0.886, with a DSe of 45.7% and a DSp of 100.0% at a cut-off of 0.917. The best-performing cross-host combination, comprising eight recombinant proteins (LprA_E, LprF_E, LpqH_E, MPB70_E, LprA_S, MPB63_S, MPB64_S, and E6C10_S), achieved an AUC of 0.991, with a DSe of 87.0% and a DSp of 100.0% at a cut-off value of 0.870.

**Table 4:** **Diagnostic performance and antigen profiles of multiple logistic regression models**

Condition	Antigen Profile	Cut-off (*)	DSe (%)	Raw AUC	Corrected AUC (95% CI)
*E. coli* only	LprA, LprF, LprG, LpqH, MPB63, MPB64, MPB70, and E6C10	0.820	80.4	0.970	0.928 (0.854–0.979)
*M. smegmatis* only	LprF, LprG, MPB63, MPB64, MPB70, and MPB83	0.917	45.7	0.886	0.836 (0.758–0.918)
*E. coli* + *M. smegmatis*	(*E. coli*) LprA, LprF, LpqH and MPB70 (*M. smegmatis*) LprA, MPB63, MPB64, and E6C10	0.870	87.0	0.991	0.955 (0.884–1.000)

DSe, Diagnostic sensitivity; DSp, Diagnostic specificity; AUC, area under the curve.

^*^The cutoff value for the MLR model was defined as the threshold corresponding to 100% specificity.

## Discussion

A major obstacle in bTB surveillance lies in the inherent limitations and logistical challenges of CMI-based assays under certain conditions. The TST, officially approved by WOAH as an antemortem test for bTB, cannot fully exclude false-positive reactions caused by NTM infections in cattle [10]. Similarly, delayed processing of blood samples for IGRA can reduce its DSe by up to 48.3% [35]. In addition, our unpublished data indicate that approximately 25% of bovine IGRA samples exhibit insufficient responses to mitogen stimulation, a proportion that can increase to nearly 70% depending on operational variables such as sample handling (e.g., transport distance) and technical factors during blood collection by veterinarians (e.g., in vitro hemolysis). Because these factors can render the interpretation of CMI-based assays inconclusive, serological assays that are less susceptible to operational variability and show high concordance with CMI-based tests may serve as valuable complementary tools for preventing bTB transmission in livestock populations. Reflecting this need, the WOAH approved the Enferplex Bovine TB antibody assay (Enfer Scientific, Naas, Ireland) in 2019 as an ancillary method for cattle with inconclusive results from CMI-based tests [36]. In a recent large-scale evaluation, the Enferplex assay, comprising 11 recombinant antigens primarily produced in *E. coli*, showed high DSp (98.4%) and over 90% DSe in PPD-boosted cattle but only 30.5% DSe in non-boosted IGRA-positive animals [14, 37]. This marked discrepancy led us to hypothesize that the absence of PTMs in *E. coli*–derived recombinant antigens may impair recognition of B-cell epitopes present in naturally infected cattle. To address this limitation, we evaluated *M. smegmatis* as an alternative expression host to identify antigen candidates that retain the practical advantages of serological assays while achieving closer agreement with IGRA outcomes.

In this study, we assessed five antigen candidates (LprA, LprF, LprG, LppX, and LpqH), which have been reported to undergo glycosylation and lipidation in *M. bovis*. These antigens were expressed in *E. coli* and *M. smegmatis* to explore whether host-dependent factors (HDFs) influence antibody reactivity. Among the ten recombinant proteins (i.e., five antigens expressed in two hosts), LprA_S achieved a DSe of 50.0% and an AUC of 0.791, significantly higher than those of its *E. coli*–derived counterpart (Table 2). This finding raises the possibility that HDFs may contribute to the preservation of conformational B-cell epitopes. In this context, glycosylation of the protein represents a biologically plausible explanation. Previous studies have demonstrated that the lipidated form of LprA is essential for the activation of antigen-presenting cells (APCs), and that approximately 60% of its predicted B-cell epitopes overlap with glycosylation sites [30, 38]. These findings underscore the potential relevance of PTMs in serological antigen screening, at least for selected antigens such as LprA. In contrast, no significant host-dependent differences in diagnostic performance were observed for LprF, LprG, LppX, or LpqH, despite previously reported PTMs in these proteins [28–30]. These results suggest that, although the glycosylated or lipidated domains of these antigens can activate APCs and promote T-cell responses via Toll-like receptor 2 signaling [39–42], host-dependent effects had only a limited impact on ROC-based discriminative performance when evaluated against IGRA status.

MPB70 and MPB83 are well-conserved MTBC proteins that have shown strong antigenicity in bTB serological tests when they are expressed in *E. coli* [13–16, 18]. To examine the influence of PTMs on antibody reactivity, we evaluated both antigens following expression in *E. coli* and *M. smegmatis*. Among the 20 single-antigen ELISAs, MPB70_E yielded the highest DSe (54.4%), consistent with its previously shown serological utility [13–16, 18]. However, MPB70_S did not further increase DSe or AUC, which likely reflects the intrinsic antigenicity of MPB70 rather than a PTM-related effect. Native MPB70 lacks known PTMs other than secretion and contains 32 experimentally validated B-cell epitopes, including an immunodominant region (residues 51–70), which likely contributes to its diagnostic performance observed across expression hosts [43]. By comparison, MPB83 is one of the few *M. bovis* proteins experimentally confirmed to be glycosylated and is predominantly secreted as a 23 kDa protein [44]. Although no statistically significant improvement in overall diagnostic performance was observed between the two forms, MPB83_S identified three IGRA-positive cattle that were missed by MPB83_E (Additional file 1). It is worth noting that one of these animals (P6) tested negative for the other 19 antigen candidates, suggesting that MPB83_S may recognize antibody subsets in IGRA-positive animals that are directed against PTM-related epitopes. This finding may be consistent with the statistically significant difference in Cohen's kappa and Fisher's exact tests when comparing MPB83_S and MPB83_E (Table 3). Given the established use of MPB83 in serological assays in multiple wildlife reservoirs, the ability of MPB83_S to identify additional IGRA-positive cattle indicates potential value for bTB monitoring in populations where TST and IGRA testing are often impractical [45, 46].

ESAT-6 and CFP-10 are well-characterized virulence factors encoded within the region of difference 1 (RD1) of *M. bovis* and are secreted via the ESX-1 (Type VII) secretion system, eliciting robust CMI responses [47]. While these antigens are highly immunogenic in TST and IGRA, their utility in bTB serological tests has been described as somewhat limited [20, 48, 49]. In this study, E6C10_S showed higher OD_450_ values in IGRA-negative cattle, resulting in compromised discriminative ability and reduced DSe compared with its *E. coli*–expressed counterpart (Table 2). This discrepancy is likely related to conformational differences, as E6C10_S exhibited altered migration and loss of His-tag detection in native PAGE and native Western blot analyses, whereas denaturing SDS-PAGE and Western blot confirmed comparable molecular weight and anti-His reactivity (Additional file 8). These findings suggest that altered epitope exposure may promote non-specific antibody binding in IGRA-negative cattle, potentially due to cross-reactive responses associated with NTM exposure, particularly given that the IGRA-negative samples analyzed in this study exhibited adequate mitogen responses. This interpretation aligns with the widespread presence of ESAT-6 and CFP-10 homologs across more than 70 mycobacterial species and with previous reports demonstrating that *Mycobacterium kansasii*–infected cattle can test positive in bPPD-based skin tests, while *M. bovis*–infected animals respond to synthetic peptides derived from *M. kansasii* ESAT-6 and CFP-10 [50–52]. Nevertheless, because these interpretations rely solely on IGRA outcomes, the extent to which the atypical antibody responses to E6C10_S reflect true bTB infection status remains uncertain. Future investigations incorporating post-mortem examination, bacterial culture, and TST will therefore be essential to clarify their diagnostic relevance.

A comprehensive interpretation of multiple statistical analyses provided additional insight into host immune responses to mycobacterial proteins. Although ROC-based analysis indicated broadly comparable diagnostic performance between antigens expressed in two hosts, these findings do not exclude the possibility that PTMs enhance bTB-relevant antigenicity, particularly given that IGRA does not always reflect true infection status. Indeed, robust antibody responses to non-protein surface antigens, such as glycolipids and glycoproteins, have been documented in animals immunized with encapsulated *M. bovis* BCG and in healthcare workers naturally exposed to *M. tuberculosis* [53, 54]. In line with these observations, our results showing higher agreement values for LprA_S, LppX_S, MPB63_S, and MPB83_S than their *E. coli* counterparts in Cohen’s kappa statistics and Fisher’s exact tests suggest that host-dependent molecular variations, potentially including PTMs, may influence antigen reactivity (Table 3). Given the essential roles of glycosylation and lipidation in mycobacterial survival and virulence, and the involvement of glycolipoproteins in APCs signaling, the immunological relevance of PTMs likely extends beyond humoral immunity to include innate and cell-mediated immune responses that modulate antibody production, warranting further integrative investigation in future studies [30, 39–42, 55, 56].

Spearman’s rank correlation analysis between age and antibody responses in IGRA-positive cattle revealed statistically significant but generally weak-to-moderate correlations for most antigens (18 out of 20; Table 3). Among the antigen candidates, MPB63 exhibited a pronounced host-dependent difference. This finding is noteworthy because MPB63 lacks well-characterized PTMs beyond secretion, and its expression in *M. smegmatis* may therefore preserve a more native-like protein conformation [26]. In this context, the differential reactivity observed for MPB63_S may reflect age-related variations in antibody recognition patterns, potentially aligning with immune profiles reported in later stages of bTB infection [57]. Similarly, the relatively stronger age-associated correlation for LprG_S than for LprG_E may indicate host-dependent variations, possibly arising from PTMs or other conformational factors. However, these interpretations should be approached with caution, as antibody responses can be detected as early as four weeks post-infection in experimental models [18, 19], and immunological stimuli such as bPPD administration may further enhance antibody reactivity irrespective of infection stage [14, 16]. Furthermore, as the infection stage was not confirmed via post-mortem examination, these findings should be interpreted within the inherent limitations of the study design. This is particularly relevant in South Korea, where stringent eradication policies restrict the progression of infection to chronic stages [10, 57]. Therefore, further studies integrating immunological observations with pathological findings are necessary to fully characterize the diagnostic utility of these antigens across different infection stages.

The MLR analysis was conducted to quantify the relative contribution of each antigen and to assess combined multi-antigen effects, after addressing potential multicollinearity (VIF < 10), and revealed synergistic interactions between antigens derived from the two expression hosts (Figure 3 and Table 4). Among the *E. coli*–derived combinations, an eight-antigen panel achieved the highest model performance (AUC: 0.970), whereas the incorporation of *M. smegmatis*–derived antigens further enhanced discriminative ability (AUC: 0.991), yielding the strongest diagnostic performance when the same number of antigens was used. The higher concordance with IGRA outcomes observed for the combined antigen model, even after bootstrap-based internal validation (AUC: 0.955), indicates that the practical advantages of ELISA, including simple sample handling, high throughput, and cost-effectiveness, may support its utility as a complementary diagnostic tool. This method can be effectively utilized in settings where immediate transport and antigen stimulation of fresh blood samples are logistically challenging, as well as in large-scale bTB surveillance programs in resource-limited environments [10, 35]. However, given the limited sample size, potential model dependency, and the imperfect concordance between IGRA results and true bTB infection status, further validation using larger independent cohorts and other diagnostic methods will be required to confirm the reliability and generalizability of these findings.

A limitation of this study is the absence of direct experimental analyses (e.g., mass spectrometry or enzymatic treatment) to characterize PTMs, including glycosylation and lipidation, in the recombinant proteins expressed in *M. smegmatis*. Although prior studies have reported such modifications for the selected antigens [28–30, 44, 58, 59], their presence remains unverified in the present study. Notably, all antigen candidates except E6C10 were naturally secreted by *M. smegmatis*, whereas their *E. coli* counterparts remained intracellular. This difference may also be relevant, as mycobacterial proteins are heterologous to *E. coli* and may not undergo native folding pathways, potentially leading to structural alterations associated with improper folding that could influence epitope accessibility. Therefore, the observed differences in antibody responses may arise from host-dependent cellular environments rather than PTMs alone. Further studies are warranted to clarify the molecular and structural determinants contributing to the elevated antibody responses observed for certain *M. smegmatis*–derived antigens.

Despite the diagnostic potential of *M. smegmatis*–derived antigens demonstrated through multiple statistical approaches, our findings should be interpreted with caution, given the absence of definitive infection confirmation by methods generally regarded as gold standards, such as bacterial culture or post-mortem examination. In practice, however, such confirmatory methods are often impractical in live animals. Under intensive bTB eradication programs, early-stage detection of *M. bovis* infection limits the identification of gross lesions at necropsy, and the sensitivity of bacterial isolation can be compromised by factors including intermittent bacterial shedding, infection stage, culture conditions, and decontamination procedures [60–63]. In the absence of a reliable gold standard for antemortem diagnosis, the TST and IGRA are officially adopted in South Korea and the European Union as reference tests for conferring bTB-free herd status and for animal movement certification [31, 64]. Accordingly, we adopted IGRA as the reference standard while implementing rigorous measures to mitigate its known limitations. To increase confidence in the negative cohort, IGRA was restricted to samples obtained from farms without reported bTB cases during the past five years, and all samples were also confirmed to be negative by MAP antibody ELISA. In addition, to minimize false-positive reactions, specific MTBC-associated synthetic peptides (ESAT-6, CFP-10, and TB10.4) were used instead of bovine PPD to reduce cross-reactivity with NTM. While these measures strengthen the reliability of our results, further studies are warranted to evaluate the robustness of our findings across different diagnostic approaches, particularly in light of known inconsistencies between diagnostic methods [10].

## Conclusion

This study suggests a potential association between PTMs and humoral immune responses relevant to bTB using *M. smegmatis*–derived recombinant antigens. Notably, *M. smegmatis*–derived LprA showed strong diagnostic potential as a single antigen, while multi-antigen combinations incorporating *M. smegmatis*–expressed proteins outperformed those based solely on *E. coli*–derived proteins. Taken together, these findings indicate the potential utility of *M. smegmatis* as an expression host for improving antigenicity in bTB serological assays.

## Supplementary Information

**Additional file 1.** **Summary of IGRA results and binary ELISA classification for each recombinant antigen.** IGRA responses were calculated by subtracting the OD values of PBS-stimulated wells from those of antigen-stimulated wells, with negative values set to zero. The prefixes P and N denote IGRA-positive (P1–46) and IGRA-negative animals (N1–N30), respectively, whereas the suffixes _E and _S indicate antigens expressed in *E. coli* and *M. smegmatis*. Individual animals were classified as positive (+) or negative (blank) for each recombinant antigen using antigen-specific OD cutoffs corresponding to 96.6% specificity, as determined by ROC curve analysis.

**Additional file 2. Purified antigen candidates expressed in both expression hosts, analyzed by SDS-PAGE.** The suffixes “_E” and “_S” denote protein expression in *E. coli* and *M. smegmatis*, respectively. Each lane was loaded with 300 ng of purified protein. SDS-PAGE was performed using 10–20% gels and stained with Coomassie Brilliant Blue. Lane 1: Protein marker (kDa); Lane 2: MPB63_E; Lane 3: MPB63_S; Lane 4: MPB64_E; Lane 5: MPB64_S; Lane 6: MPB70_E; Lane 7: MPB70_S; Lane 8: MPB83_E; Lane 9: MPB83_S; Lane 10: LprA_E; Lane 11: LprA_S; Lane 12: LprF_E; Lane 13: LprF_S; Lane 14: LprG_E; Lane 15: LprG_S; Lane 16: LppX_E; Lane 17: LppX_S; Lane 18: Protein marker (kDa); Lane 19: LpqH_E; Lane 20: LpqH_S; Lane 21: E6C10_E; Lane 22: E6C10_S.

**Additional file 3. Expression conditions for recombinant proteins in *E. coli* and *M. smegmatis*.** Host strains, expression vectors, culture media, incubation conditions, antibiotic selection, isopropyl β-D-1-thiogalactopyranoside (IPTG) concentrations, and protein localization for each recombinant protein evaluated in this study are summarized. “Not applicable” indicates conditions that are not relevant to the respective expression system.

**Additional file 4. Primer sequences used for cloning target genes from *M. bovis* BCG Tokyo 172 genomic DNA for recombinant expression in *E. coli* and *M. smegmatis*.** Restriction enzyme recognition sites incorporated into the primers for cloning are underlined, and the corresponding enzyme names are indicated in parentheses at the end of each sequence. For certain genes, codon-optimized synthetic constructs obtained from GenScript were used as templates. The Forward 2 and Reverse 2 primers for LprA and LppX were designed to introduce a specific point mutation (GGATCC → GGGTCC) into the vector-encoded BamHI site.

**Additional file 5. Summary of logistic regression analysis (*E. coli*).**

**Additional file 6. Summary of logistic regression analysis (*M. smegmatis*).**

**Additional file 7. Summary of logistic regression analysis (*E. coli* + *M. smegmatis*).**

**Additional file 8. Characterization of ESAT-6 and CFP-10 fusion protein under native and denaturing conditions. (A) **Native-PAGE was performed using 10–20% gels and stained with Coomassie Brilliant Blue. Each lane was loaded with 1 µg of purified protein. Lane 1: E6C10_E; Lane 2: E6C10_S. **(B)** Western blotting was performed using anti-6X His-tag antibody under the native conditions. Lane 3: E6C10_E (300 ng); Lane 4: E6C10_S (300 ng); Lane 5: E6C10_E (500 ng); Lane 6: E6C10_S (500 ng); Lane 7: E6C10_E (1 ug); Lane 8: E6C10_S (1 µg) **(C)** Western blotting was performed using anti-6×His-tag antibody under the denaturing condition. Lane 9: E6C10_E (300 ng); Lane 10: E6C10_S (300 ng). Antigens expressed in *E. coli* and *M. smegmatis* are denoted by the suffixes “_E” and “_S,” respectively.

## Data Availability

All data generated or analysed during this study are included in this published article and its Additional files.
